# Role of micronutrients in the modulation of immune system and platelet activating factor in patients with COVID-19; a narrative review

**DOI:** 10.3389/fnut.2023.1207237

**Published:** 2023-09-15

**Authors:** Saeid Doaei, Afrouz Mardi, Maryam Zare

**Affiliations:** ^1^Department of Community Nutrition, Faculty of Nutrition Sciences and Food Technology, National Nutrition and Food Technology Research Institute, Shahid Beheshti University of Medical Sciences, Tehran, Iran; ^2^Department of Public Health, School of Health, Ardabil University of Medical Sciences, Ardabil, Iran; ^3^Department of Nutrition, Khalkhal University of Medical Sciences, Khalkhal, Iran

**Keywords:** COVID-19, platelet activating factor, immunity, micronutrient, vitamin, mineral

## Abstract

**Background:**

Dietary micronutrients may play important roles in the improvement of the immune responses against SARS-CoV-2. This study aimed to assess the effect of micronutrients on platelet activating factor (PAF) and immunity with a special focus on the coronavirus disease 2019 (COVID-19).

**Methods:**

All paper published in English on the effects of micronutrients including fat soluble vitamins, water soluble vitamins, and minerals on PAF, immunity, and COVID-19 were collected from online valid databases.

**Results:**

Vitamin A may modulate the expression of PAF-receptor gene in patients with COVID-19. Vitamin D may modulate inflammatory response through influencing PAF pathway. Vitamin E may improve COVID-19 related heart injuries by exert anti-PAF activities. Vitamin C status may have PAF related anti-inflammatory and micro-thrombotic effects in SARS-CoV-2 patients. Furthermore, some trace elements such as copper, selenium, and iron may have key roles in strengthens immunity by inactivate PAF acetyl hydrolase.

**Conclusion:**

This narrative review study highlighted the importance of micronutrients in the improvement of immune function through modulation of PAF in patients with COVID-19. Further longitudinal studies are warranted.

## Introduction

Coronavirus disease 2019 (COVID-19) is a viral infectious disease that caused a worldwide health crisis (1). So far, more than six million people have died from the disease (2). The symptoms of this disease are non-specific such as fever, cough, dyspnea, severe muscle pain, diarrhea, dysregulation of some hematological parameters, severe pneumonia, pulmonary micro thromboses, and in some cases can lead to death (3–5). SARS-CoV-2 may be colonized in the respiratory tract, but it may attack the gastrointestinal tract, neurological system, and kidneys (6, 7).

Severe acute respiratory syndrome coronavirus 2 (SARS-CoV-2) was reported to utilize angiotensin converting enzyme II (ACE2) for entering into lymphocyte, monocyte, pulmonary alveolar, and esophageal epithelial cells. Rapid virus replication leads to cytokine storms in patients with severe COVID-19 (8, 9). Moreover, SARS-CoV-2 is connected to a pre-thrombotic state through the action of various mediators, such as platelet activating factor (PAF) (10). PAF is synthesized by several cell types including platelets, polymorphonuclear cells, endothelial cells, and monocytes/macrophages. PAF is a powerful mediator of inflammation proinflammatory cytokines, immune responses, and free radicals may induce PAF synthesis (11, 12). PAF has crucial roles in immune reactions such as inhibition of T cell proliferation and IL-2 production in response to mitogens (13). PAF is highly neurotoxic and can cause neuronal injury and death (14).

The spike proteins of SARS-CoV-2 stimulates the production of PAF in monocytes. Cells producing more PAF such as mast cells in the lungs can increase the inflammatory response in the patients with COVID-9 and exacerbate the clinical condition (15, 16). PAF, as one of the potent phospholipid mediators with a wide range of bioactivity, has been shown to increase the binding of pathogenic bacteria to pharyngeal epithelial cells. The expression of PAF receptor genes was reported to be up-regulated in COVID-19 which may play an important role in the binding and entry of the pathogen into the cells (17). It is well established that nutritional status plays an important role in immune function. It is reasonable to consider the role of dietary composition in the risk of COVID-19 (18, 19). Adequate dietary intake is important for the development of immune responses and following a well-balanced diet in combination with certain dietary supplements may strengthen and optimize the function of the immune system (20). Micronutrient deficiencies affect both innate and adaptation immunity, causing some persons to be more susceptible to infections. Some micronutrients including several vitamins (e.g., vitamins A, D, E, K, C, and B-complex) and trace elements (e.g., zinc, selenium, copper, magnesium, and iron) are reported to play a key role in supporting the immune system through several mechanisms such as anti PAF effects. The deficiency of these nutrients may promote the infectious diseases (21, 22). A healthy diet can potentially inhibit such actions and exert protective actions against SARS-CoV-2 and its accompanying pathological entities, such as thrombosis (23).

Due to lack of enough information on the effects of micronutrients on PAF and immunity, this narrative review aimed to investigate the effects of micronutrients on (PAF) and immunity in patients with COVID-19.

## Methods

All papers published in English from 1990 to 2023 regarding clinical trials on the effects of micronutrients including fat soluble vitamins, water soluble vitamins, and minerals on COVID-19, immunity, PAF were collected from online valid databases using the following keywords: “COVID-19 or SARS-CoV-2 or coronavirus or immunity or immune system” AND “platelet activating factor or PAF” AND “micronutrient or vitamin or mineral or retinol or retinoic acid or calciferol or calcitriol or tocopherol or phylloquinone or thiamin or riboflavin or niacin or nicotinamide or pyridoxine or folate or folic acid or cobalamin or biotin or ascorbic acid or calcium or iron or zinc or selenium or copper.”

### Fat-soluble vitamins and COVID-19

#### Vitamin A

Vitamin A is essential for normal growth and development, maintenance of the natural structure of epithelial tissue and integrity of the immune system, and may inhibit viral replication (24). In the respiratory system, vitamin A has a special function in reducing harmful inflammation, promoting respiratory epithelium healing, and preventing fibrosis (25). Vitamin A plays an important role in regulating the number and function of NK cells, macrophages, and neutrophils (26). Furthermore, vitamin A has an up regulatory role in the early stages of NK cells differentiation by reducing interferon (IFN) γ expression and increasing the secretion of IL-5. Vitamin A was also reported to increase the secretion of cytokines IL-12 and IL-23 (22). Moreover, vitamin A can modulate the gene expression of PAF-receptor (PAFR) (23) and the PAF-PAFR pathway is regulated by a tissue-specific response to vitamin A (27). The ability of the PAF-PAFR signaling to induce a robust systemic pro-inflammatory and delayed immune suppressive responses was previously reported in various pathological conditions (28). Interestingly, vitamins A may prevent from entering the virus in the bloodstream from the intestinal tract by changing the intestinal microbiome to Lactobacillus species (29). Vitamin A supplementation may up-regulate the immune system in fight against SARS-CoV-2 infections (30). Vitamin A supplementation demonstrated efficacy in improving the inflammatory response in patients with SARS-CoV-2 (31) and decrease the severity of disease in ICU-admitted patients with COVID-19 (32, 33).

#### Vitamin D

Vitamin D receptors are largely expressed in the epithelium of the respiratory system and the macrophages (34). Vitamin D strengthens the epithelium and increases the synthesis of antimicrobial peptides including Cathelicidin, IL-37, and defensins in the epithelium and macrophages. A recent study showed that the presence of 1, 25 dihydroxy calciferol increased the production of macrophage catalysts such as IL-37, inhibits the response induced by Th1, IL-2, and IFN-γ, improved regulatory induction of T cells, and caused inhibited inflammatory processes (35). Vitamin D enhances innate immunity with phagocytosis, the conversion of monocytes to macrophages, and enhances cellular immunity by suppressing the cytokine storm with effects on IFN-γ and TNF-α (35, 36). Vitamin D ay have inhibitory effects on the replication of coronavirus and has antiviral activity in the respiratory tract (35, 37). Vitamin D is involved in reducing the infection severity and mortality of patients with SARS-CoV-2. In addition, vitamin D reduces cytokine storm and regulates adaptive immunity by inhibiting T helper and stimulating T cell induction. Vitamin D reduces the expression of pro-inflammatory cytokines and increases the expression of anti-inflammatory cytokines by macrophages (38). Moreover, vitamin D may modulate inflammatory response through the PAF pathway (23). More specifically, vitamin D may reduce the activity of PAF biosynthetic enzymes and increases the activity of its catabolic enzymes (39). Vitamin D may attenuate PAF signaling via the PAF-R in SARS-CoV-2 infection (40).

One study reported that 5000 IU/day of oral vitamin D supplementation for 2 weeks reduces the time to recovery for cough and gustatory sensory loss among patients with mild to moderate SARS-CoV-2 symptoms (41). Furthermore, vitamin D supplementation at an oral dose of 200,000 to 300,000 IU combined with other nutrients for 1 week strengthened the immune system against SARS-CoV-2 (30). Another study provided suggestive evidence on the association between higher predicted circulating 25(OH)D concentrations and a lower risk of SARS-CoV-2 infection. Greater intake of vitamin D supplements was associated with a lower risk of hospitalization (42). Vitamin D in a dose of 1000–2000 IU/day was reported to be required during the outbreak of SARS-CoV-2 (43). Vitamin D was related to reduction in the rate of mortality and morbidity by decreasing the levels of D-dimer, TNF-α, and IL-6 in patients with SARS-CoV-2 (44). Oral 25(OH)D_3_ was able to correct vitamin D deficiency/insufficiency in patients with COVID-19 that resulted in improved immune function by increasing blood lymphocyte percentage (45).

The early administration of high dose versus standard-dose vitamin D3 to at-risk older patients with COVID-19 improved overall mortality at day 14. The effect was no longer observed after 28 days.

#### Vitamin E

Vitamin E is a biological antioxidant which protect cell membranes from oxidative damage and enhances immune function (46). There is a negative relationship between serum level of vitamin E and the risk of infection in adults (47). Vitamin E protects cell membranes from free radicals and plays an important role in increasing the production of NK cells and interleukins (34). Also, vitamin E was reported to increase T lymphocyte-mediated immune function in response to mitogens and IL-2 (48). The results of another study indicated that vitamin E supplementation (200 mg/day) increased neutrophil chemotaxis and phagocytosis, NK cell activity, and mitogen dependent proliferation (49). Vitamin E supplementation (200 IU/day) for 1 year reduced the risk of respiratory infections in adults (50). However, another study reported that vitamin E supplementation of 200 mg/d has no effect on the severity of respiratory infections in the elderly (51). The amount of PAF production in the vitamin E-deficient people was reported to be twice higher than people with vitamin E supplementation. Increasing of PAF production in vitamin E deficiency was attributed to increased synthesis and/or decreased catabolism of PAF (52). Alpha-tocopherol may also have anti-PAF activity (53, 54).

#### Vitamin K

In severely affected COVID-19 patients, for instance, the massive recruitment and activation of neutrophils is responsible for the profuse release of elastases and other proteolytic enzymes which cause the irreversible degradation of elastic fibers. A significant correlation was observed between dp-ucMGP matrix Gla protein (MGP) desphospho-uncarboxylated (dp-uc; i.e., inactive) and the degree of fiber elastic degradation in patients with SARS-CoV-2. High plasma dephosphorylated uncarboxylated matrix Gla-protein (dp-ucMGP) concentrations reflect a low vitamin K status and dp-ucMGP is a biomarker of functional vitamin K status. Fiber elastic degradation degradation is associated with increased proteolytic activity in the lungs of patients with SARS-CoV-2, may lead to up-regulation of vitamin K-dependent MGP synthesis and extrahepatic depletion of vitamin K stores. Impairment of circulatory activation of MGP degrades the elasticity of the fibers and increases the circulating level of dp-ucMGP. Degradation of lung elastic fibers in patients with SARS-CoV-2 is associated with pneumonia, COPD, cystic fibrosis, and bronchiectasis (55–58). The level of decreased serum level of vitamin K in patients with SARS-CoV-2 is associated with a poor prognosis (59, 60).

### Water-soluble vitamins and COVID-19

#### Vitamin C

Vitamin C as an antioxidant eliminates reactive oxygen species (ROS) and protects biomolecules such as proteins, lipids, and nucleotides from oxidative and functional damage (61). Other functions of vitamin C include iron absorption and support of the immune system (24). Vitamin C has some roles in leukocyte invasion to infectious sites, leukocyte phagocytosis, supporting lymphocyte T cell function (production CD8+), and antibody production (62). Furthermore, vitamin C has positive effects on the symptoms and duration of respiratory infections, duration of viral infections, and prevents pneumonia (63–65).

Inhibition of PAF formation by vitamin C was reported in a previous study on cardiovascular disease (66). PAF may contribute to cerebral ischemia-reperfusion injury by increasing the generation of oxygen radicals (67).

Furthermore, vitamin C reduces oxidative stress (68), which is a strong trigger for synthesis of PAF (69), and its receptor (70). Vitamin C status may thus affect the inflammatory and micro-thrombotic status through medulation of PAF level (23).

In SARS-CoV-2, pro-inflammatory cytokines, IL-1β and TNF-α, increased rapidly after infection and cause more secretion of IL-6 and IL-8, which promotes the pro-inflammatory condition. Vitamin C has been suggested to combat against cytokine storm during SARS-CoV-2 infections. Vitamin C reduces the levels of pro-inflammatory cytokines, including TNF-α, and increases the anti-inflammatory cytokine IL-10. IL-10 has an anti-inflammatory effect and controls inflammation in SARS-CoV-2 by decreasing IL-6 (61). Vitamin C prevents excessive inflammatory activity in lymphoid and myeloid cells and high doses of vitamin C act as a pro-oxidant for immune cells and an antioxidant for pulmonary epithelial cells. In humans, vitamin C supplementation improves the immune system and reduces the severity and duration of infectious diseases (71, 72). Studies have shown that vitamin C supplementation may control respiratory tract infections and reduces the mortality of patients with infectious diseases (73). Vitamin C supplementation (from 50 to 1500 mg/kg/d) can be considered as a complementary treatment for SARS-CoV-2 (30, 74–76). Therapeutic doses of vitamin C (24 g/d intravenously for 7 days) were also assessed in critically ill patients with COVID-19. This study found no significant difference in mortality and duration of mechanical ventilation, but reported improvements in oxygenation (the ratio of arterial partial pressure of oxygen to fraction of inspired oxygen [PaO2/FIO2]) (77). However, there is no evidence at this time point to support intravenous high-dose vitamin C in the management of COVID-19 (78). Vitamin C 1000 mg once daily had no effect on disease progression in mild – to – moderate patient with COVID-19 (79). The high-dose intravenous vitamin C failed to improve invasive mechanical ventilation-free days in 28 days, but might show a potential signal of benefit in oxygenation for critically ill patients with COVID-19 improving PaO2/FiO2 even though (80).

#### B- group vitamins

B vitamins regulate inflammatory response and intestinal immunity and play an important role in proliferation and evolution of lymphocytes, which are an important part of the primary immune response (30, 61).

#### Vitamin B1 (thiamine)

This vitamin is a coenzyme for the metabolism of carbohydrates and different types of amino acids and is also involved in energy metabolism (24). Thiamine deficiency affects the immune system, enhancing the inflammation of the nerves and increasing the inflammatory and antibody responses. Thiamine deficiency causes inadequate antibody responses and worsens the symptoms. Administration of high-dose vitamin B1 in the early stages of SARS-CoV-2 disease was reported to reduce hospitalization and hypoxia symptoms (61, 81, 82).

#### Vitamin B2 (riboflavin)

Vitamin B2 is responsible for releasing energy from macronutrients, the conversion of pyridoxine (B6) to pyridoxal phosphate, and the biosynthesis of vitamin B3 from tryptophan (24). Riboflavin (35 mL, 500 μmol/L) Combined by UV radiation irreversibly destroys nucleic acids, such as DNA and RNA and pathogenic microorganisms are no longer able to replicate. Riboflavin and UV rays have been reported to be effective against MERS-COV and possibly against SARS-CoV-2 (83, 84).

#### Vitamin B3 (nicotine amide, niacin)

Nicotinamide mainly acts as a component of two coenzymes, namely nicotinamide adenine dinucleotide (NAD) and nicotinamide adenine dinucleotide phosphate (NADP). NAD and NADP coenzymes are involved in the metabolism of carbohydrates, fats, proteins, and alcohol (24). A dose of 2000 mg/day reduced neutrophil infiltration and pro-inflammatory cytokines such as IL-6. Nicotinamide may reduce viral replication and strengthens the body’s defense mechanism (61, 84). The results of recent studies indicated that NAD+ may improve phosphoribosyl pyrophosphate (PRPP) to help innate immunity in patients with SARS-CoV-2 (82, 85).

#### Vitamin B6 (pyridoxine, pyridoxal, and pyridoxamine)

Vitamin B6 helps in the synthesis, catabolism, and transport of amino acids and the conversion of essential amino acids to other amino acids. Vitamin B6 has a protective role in cellular immunity (24, 86). Vitamin B6 up-regulates interleukin-10 and immunosuppressive cytokines, inactivates macrophages and monocytes, inhibits antigen-expressing cells and T cells, and decrease the inflammatory effect of IL-6 and TNF-α (82). Vitamin B6 deficiency causes the atrophy of the spleen and thymus, a decrease in the number of T lymphocytes, impairment of lymphocyte proliferation, and a reduction in the T lymphocyte-mediated immune response (87). Patients with SARS-CoV-2 often respond to the virus by increased T cell responses and secretion of pro-inflammatory cytokines. Pyridoxal phosphate supplements reduce the symptoms of SARS-CoV-2 by regulating the immune response, reducing pro-inflammatory cytokines, strengthening endothelium, and preventing blood clotting (82).

#### Vitamin B9 (folic acid, folate)

Folic acid is an essential vitamin for DNA synthesis and may improve the adaptive immune response (82). Folic acid deficiency in animals causes the atrophy of the spleen and thymus and reduces the number of circulating T lymphocytes (47). Folic acid has been recently shown to inhibit the function of furin (an enzyme associated with viral and bacterial infections) through binding to the SARS-CoV-2 spike protein. Furthermore, Folic acid may prevent virus entry into the cell and can be useful in the early stages of SARS-CoV-2 (82). The coronavirus polypeptide encodes two proteases, 3-C-like protease (M-pro) and Papain-like protease (PL-pro), both of which are targets for drug detection in SARS and MERS coronavirus. Folic acid has a strong potential to form hydrogen bonds with the active sites of M-pro in SARS-CoV-2 and therefore be a possible therapeutic strategy (48).

#### Vitamin B12 (methyl cobalamin, adenosyl cobalamin, and hydroxy cobalamin)

Vitamin B12 is a stimulator for the formation of red blood cells, synthesis of nucleic acids and nucleoproteins, and metabolism of nerve tissue (24). Vitamins B12 and B6 and folic acid affect the activity of NK, cytotoxicity of T lymphocytes, and fight against viruses (46). Vitamin B12 deficiency is associated with increased oxidative stress, lactate dehydrogenase, blood coagulation activation, and vascular contractions (82). One study found that methylcobalamin supplementation reduced tissue damage and SARS-CoV-2-related symptoms (88). In another study, patients with SARS-CoV-2 who received vitamin B12 (500 μg), vitamin D (1000 IU), and magnesium (150 mg) supplements had reduced symptoms, disease severity, and need for oxygen (89).

### Minerals and COVID-19

#### Zinc (Zn)

Zinc is a cofactor of more than 100 different enzymes and plays an important role in the immune system (24). There are several studies on the role of zinc in several biological processes including the provision and development of cellular immunity and generating innate and acquired immune responses to viral infections (84, 90, 91). As an antioxidant, zinc protects the body against viral and bacterial infections. *In vitro* studies have shown that zinc deficiency alters the function of inhibitory cells in pulmonary epithelial tissues through the up-regulation of IFN-γ and TNF-α. Zinc deficiency also reduces the number of lymphocytes and impairs their function, while supplementation of zinc increases the number of T and NK cells, and IL-2. Zinc deficiency impairs the immune function, especially the humoral and cellular immune function, and spreads infectious diseases. Furthermore, zinc deficiency increases the production of pro-inflammatory and inflammatory cytokines, causes impairment of immunity, reduced B lymphocyte count, and lower antibody production. The role of zinc in the prevention of respiratory infectious diseases is also important (30, 61, 92, 93). Recent studies have shown that increased intracellular zinc concentrations inhibit the function of RNA polymerase, which is required for virus RNA replication, and promote adaptive immunity (30, 84).

A low zinc diet reduces platelet aggregation suggesting a role of zinc in hemostasis (94). Zinc and copper chelate complexes are reported to have a PAF inhibitory activity mainly attributed to stereo chemical interactions (95).

Recent meta-analyses have shown that zinc reduces mortality in acute pneumonia and inhibits of replication SARS-CoV-2. Zinc also prevents SARS-CoV-2 synthesis, replication, and transcription (30, 96–100). Zinc supplementation (200 mg/d) not only affect the virus replication, but also affect SARS-CoV-2-related symptoms, such as diarrhea and respiratory tract infections (84, 101). One study found that serum zinc concentrations in patients with SARS-CoV-2 were lower than in healthy individuals (100). Recent research indicated that zinc supplementation for 11 days on average improved oxygen saturation (102).

The combination of doxycycline and zinc had a protective effect in patients with SARS-CoV-2 infection (103). High doses of zinc gluconate, ascorbic acid, or a combination of zinc gluconate and ascorbic acid did not significantly shorten the duration of symptoms associated with the virus compared with the control group (104).

#### Magnesium (Mg)

Magnesium is considered a cofactor of more than 300 enzymes and is involved in energy metabolism, protein synthesis, immunity, and metabolism (24, 81). Chelating agents such as Mg^2+^ are reported to reduce the activity of PAF biosynthetic enzymes, such as Lyso-PAF acetyltransferase (105). Magnesium deficiency reduces the activity of immune cells and increases cytokine storm in patients with SARS-CoV-2 (82). One study found that magnesium deficiency decreased lung function (106). Magnesium has some effects on pulmonary dilation and release of acetylcholine and histamine which can lead to anti-inflammatory effects (81, 107, 108). Injection of 680 mg/d of magnesium supplementation improved respiratory symptoms in adults with SARS-CoV-2 (109).

#### Copper (Cu)

Copper plays an important role in respiration, immune function, and defense against free radicals in human body. Copper is also required as the enzyme Cu-Zn superoxide dismutase for immunological responses against infection and oxidative phosphorylation. Copper supports the function of neutrophils, monocytes, macrophages, and NK cell activity. Human studies have shown that people with low dietary copper intake have reduced lymphocyte proliferation and IL-2 production (75). Copper has antiviral properties, such as inactivation of DNA or RNA virus, destruction of the virus genome, and blockage of proteases (101). Moreover, copper plays an important role in the regulation of IL2, which plays an important role in the proliferation of T-helpers and the balance between Th1 and Th2, and the cytotoxicity of NK cells (101, 102, 105, 106).

#### Selenium (Se)

Selenium acts as a cofactor for the glutathione peroxidase (GP) enzymatic complex which acts as an antioxidant (24). Selenium is a key element in the immune system and lowers the level of cytotoxicity and inflammation in lung tissues in viral infections (37). Free selenium in serum has a significantly positive effect on the pathogenicity of viral infections. Selenium may help alter the genetic expression pattern of the virus (110). Low concentrations of selenium in humans are associated with decreased NK cell activity and increased mycobacterium disease. Selenium deficiency increases mutations in viruses (111–113). Dietary selenium deficiency causes oxidative stress in the host and under oxidative stress conditions, changes the viral genome from normal to pathogenic, causing mutations in the virus genome, and virus transformation. Selenium deficiency not only disrupts the immune system, but also leads to rapid mutations in the RNA virus (84).

Furthermore, selenium deficiency caused increased PLD activity and PA production which led to enhanced Lyso-PAF-AcT activity and an overall increase in PAF production. The regulation of Lyso-PAF-AcT activity by Selenium may constitute an important control mechanism for the maintenance of PAF levels in endothelial cells (114). Selenium has a special role in blocking the ACE2 receptors and this mechanism plays an important role against SARS-CoV-2. A potential benefit from improved selenium intake was reported among individuals consuming a low-selenium diet to mitigate COVID-19 severity (115). Based on different studies, selenium supplementation from 100 to 300 μg/d improves immune function in humans (26, 116, 117). However, a recent study recommended against high-dose of selenium supplementation and only support vitamin supplementation that compensate selenium deficiency (19). The worse outcomes of SARS-CoV-2 on the grounds of selenium deficiency may be at least in part attributed to increased PAF, an associated pro-thrombotic state, reducing NK cell activity, and increased mutations in the RNA virus.

#### Iron (Fe)

Iron is also a component of myoglobin, transferrin, and oxidative enzymes. Iron is very important for preventing nutritional anemia and plays a major role in respiration and developing immunity (24). Iron deficiency reduced T lymphocyte production and caused respiratory infections and affected function cellular immune. Therefore, iron is recommended at 8 mg/d for men and 18 mg/d for women in their reproductive periods (118). Iron can help to the production of hydroxyl radicals, which help neutrophils kill viruses and bacteria. Of course, microorganisms grow to need iron (46, 58). For patients with SARS-CoV-2 who are iron deficient, the length of hospitalization is longer than the others (119). However, higher doses of iron lead to oxidative stress, impaired immune function, and inflammatory lesions and provide a favorable environment for viral mutations.

### Recommendations

There is no proven dietary recommendation to prevent coronavirus. However, since the host metabolic status influences the progression of the disease, a healthy balanced diet should be followed. A healthy diet as promoted by international bodies ensures the provision of micronutrients such as vitamins A, C, D, E, and B complex, selenium, iron, zinc, copper, which play a role in the immune system through modulation of PAF action and metabolism, is strongly recommended (23). A high-quality dietary pattern is associated with reduced risk of COVID-19 infection and hospitalization rates (120, 121). A healthy diet containing PAF inhibitors may target both inflammation and thrombosis and prevent the deleterious effects of COVID-19 (74, 122). A diet rich in micronutrients is highly advised to prevent deficiencies. However, supplementation of high doses of micronutrients above the RDAs is not yet supported (123).

## Conclusion

Exposure to contaminants, malnutrition, and other factors affect the immune system. Some micronutrients improve the function of the immune system to protect against many diseases such as SARS-CoV-2 It is necessary to consume enough micronutrients through healthy diet which play an important role in strengthening the immune system. Understanding the supporting roles of micronutrients in immune system can play an important role in the prevention of infection to SARS-CoV-2 and so the management of patients with SARS-CoV-2. The present study highlights the importance of promoting healthy diet and micronutrients as a therapy, coupled with pharmacological treatment for the prevention, severity reduction, and boost recovery from SARS-CoV-2 and the post- SARS-CoV-2 syndrome. A healthy diet may target both inflammation and thrombosis and prevent the deleterious effects of SARS-CoV-2. Future RCT studies are warranted to identify the effects of a healthy diet and micronutrients on improving the status of patients with SARS-CoV-2 (Table 1).

**TABLE 1:** The characteristics of randomized clinical trials on the effects of micronutrients against SARS-CoV-2.

Micronutrient	References	Study population	Settings	Outcome
Vitamin A	Beigmohammadi et al. (32)	60	Iran	Supplementation with vitamins A, B, C, D, and E could improve the inflammatory response and decrease the severity of disease in ICU-admitted patients with SARS-CoV-2
	Rohani et al. (31)	182	Iran	Vitamin A supplementation improved some clinical and paraclinical symptoms in patients with SARS-CoV-2
	Somi et al. (33)	30	Iran	The results of this pilot clinical trial showed no benefit of vitamin A compared with the common treatment on outcome severity in hospitalized patients with SARS-CoV-2
Vitamin D	Sabico et al. (41)	69	Saudi Arabia	A 5000 IU daily oral vitamin D3 supplementation for 2 weeks reduces the time to recovery for cough and gustatory sensory loss among patients with sub-optimal vitamin D status and mild to moderate COVID-19 symptoms.
	Maghbooli et al. (45)	106	Iran	Our analysis indicated that oral 25(OH)D3 was able to correct vitamin D deficiency/insufficiency in patients with SARS-CoV-2 that resulted in improved immune function by increasing blood lymphocyte percentage.
Vitamin C	Zhang et al. (80)	56	China	The high-dose intravenous vitamin C failed to improve invasive mechanical ventilation-free days in 28 days, but might show a potential signal of benefit in oxygenation for critically ill patients with SARS-CoV-2 improving PaO2/FiO2 even though.
	Fogleman et al. (79)	98	US	Vitamin C 1000 mg once daily has no effect on disease progression.
Zinc	Stambouli et al. (103)	172	Tunisia	The combination of doxycycline and zinc has a protective effect in patients with SARS-CoV-2 infection.
	Thomas et al. (104)	214	US	Treatment with high-dose zinc gluconate, ascorbic acid, or a combination of the 2 supplements did not significantly decrease the duration of symptoms compared with standard of care.

## Author contributions

All authors designed the study, involved in the data collection, analysis, drafting of the manuscript, design of the study, and analysis of the data, critically reviewed the manuscript, and read and approved the final manuscript.
